# Synergistic effect of cisplatin and synchrotron irradiation on F98 gliomas growing in nude mice

**DOI:** 10.1107/S0909049513016567

**Published:** 2013-07-03

**Authors:** Clement Ricard, Manuel Fernandez, Herwig Requardt, Didier Wion, Jean-Claude Vial, Christoph Segebarth, Boudewijn van der Sanden

**Affiliations:** a INSERM U836, Grenoble Institut des Neurosciences, Grenoble, France; b Université Joseph Fourier, Grenoble, France; c European Synchrotron Radiation Facility, Grenoble, France; d CNRS UMR 5588, Laboratoire Interdisciplinaire de Physique, St Martin d’Hères, France

**Keywords:** synchrotron photoactivation therapy, cisplatin, glioma, two-photon microscopy, synergistic effects

## Abstract

Among brain tumors, glioblastoma multiforme appears as one of the most aggressive forms of cancer with poor prognosis and no curative treatment available. Recently, a new kind of radio-chemotherapy has been developed using synchrotron irradiation for the photoactivation of molecules with high-*Z* elements such as cisplatin (PAT-Plat). This protocol showed a cure of 33% of rats bearing the F98 glioma but the efficiency of the treatment was only measured in terms of overall survival. Here, characterization of the effects of the PAT-Plat on tumor volume and tumor blood perfusion are proposed. Changes in these parameters may predict the overall survival. Firstly, changes in tumor growth of the F98 glioma implanted in the hindlimb of nude mice after the PAT-Plat treatment and its different modalities have been characterized. Secondly, the effects of the treatment on tumor blood perfusion have been observed by intravital two-photon microscopy. Cisplatin alone had no detectable effect on the tumor volume. A reduction of tumor growth was measured after a 15 Gy synchrotron irradiation, but the whole therapy (15 Gy irradiation + cisplatin) showed the largest decrease in tumor growth, indicating a synergistic effect of both synchrotron irradiation and cisplatin treatment. A high number of unperfused vessels (52%) were observed in the peritumoral area in comparison with untreated controls. In the PAT-Plat protocol the transient tumor growth reduction may be due to synergistic interactions of tumor-cell-killing effects and reduction of the tumor blood perfusion.

## Abstract 2

Synchrotron photoactivation therapy of cisplatin relies on a synergistic effect of synchrotron X-rays and platinum and leads to tumor-cell-killing effects and reduction of the tumor blood perfusion.

## 1. Introduction   

Radiotherapy has increased the overall survival of patients with primary brain tumors; however, the dose is limited by the radiosensitivity of the surrounding tissues (Schultheiss *et al.*, 1995 ▶). A total dose of 60 Gy, delivered in 30 fractions of 2 Gy, is the maximum dose recommended for the treatment of glioblastoma multiforme. This dose is too low to sterilize the tumor that inevitably recurs.

Chemotherapy effects are generally poor as the drug delivery to the tumor is difficult (Muldoon *et al.*, 2007 ▶). The blood-brain barrier (BBB), despite its disruption in some part of the tumor, and the interstitial fluid pressure are the main obstacles to the free diffusion of therapeutic molecules. Moreover, tumor cells often migrate in healthy surrounding tissues at the distance of the original lesion where the BBB is still intact. In spite of the drawbacks of radiotherapy and chemotherapy, a combination of both have improved the survival of patients (Bartelink *et al.*, 2002 ▶; Stupp *et al.*, 2005 ▶).

Another approach to ameliorate the percentage of survivors may be to increase the local dose deposition during radiotherapy using X-ray photoactivatable drugs. This chemo-radiotherapy was successfully tested at the European Synchrotron Radiation Facility (ESRF) (Biston *et al.*, 2004 ▶; Adam *et al.*, 2003 ▶). This method, synchrotron stereotactic radiotherapy, consists of a tomographic irradiation of the tumor volume with kilo-voltage synchrotron X-rays after loading the tumor with high-*Z* compounds such as iodine (Adam *et al.*, 2003 ▶, 2006 ▶) or platinum (Biston *et al.*, 2004 ▶). An irradiation of a high-*Z* compound around its *K*-edge results merely in a photoelectric effect generating photoelectrons. Cisplatin [*cis-*diamminedichloroplatinum(II)] is a chemotherapeuthic drug that is used in the treatment of solid tumors and forms adducts with DNA (Boulikas & Vougiouka, 2003 ▶). Biston *et al.* (2004 ▶) demonstrated that rats bearing F98 gliomas were cured after the intratumoral injection of cisplatin followed 24 h later by synchrotron irradiation. In that study, survival analyses were performed, whereas the aim in the present study is to understand and to analyse underlying tumor morphological and physiological changes that determine survival, such as volume and blood perfusion at the presence and absence of the photoelectric effects. Changes in these parameters were measured in time after different treatment modalities on F98 gliomas growing in the hindlimb of nude mice. This heterotopic glioma model was preferred over an orthotopic glioma model, because tumor volume changes can be easily evaluated and intravital two-photon microscopy observations (Helmchen & Denk, 2005 ▶; Denk *et al.*, 1990 ▶) of modifications in tumor blood perfusion in peritumoral areas can be accomplished.

## 2. Materials and methods   

### 2.1. Animal care guidelines   

In accordance with the policy of Grenoble Institute of Neuroscience and the French legislation, experiments were performed in compliance with the European Community Council Directive of 24 November 1986 (86/609/EEC). The research on animals was authorized by the Direction Départementale des Services Vétérinaires de l’Isère, Ministère de l’Agriculture et de la Pêche, France and the Direction Départementale de la protection des populations, Préfecture de l’Isère-France (B. van der Sanden, PhD, permit number 38 09 40). Five-week-old female nude mice (*n* = 146; Charles River Laboratories, France) were housed in cages with food and water *ad libitum* in a 12 h light/dark cycle at 295 ± 1 K.

### 2.2. Tumor implantation   

Animals were briefly anesthetized with inhalation of isoflurane (4%) in air. 50 µl of a solution of 10^5^ F98 glioma cells in DMEM was slowly injected subcutaneously in the left hindlimb of the animal. Animals were housed under standard conditions for 2.5 weeks or 3.5 weeks in order to obtain tumors with a mean volume of 106 ± 11 mm^3^ (small-volume group) and 480 ± 33 mm^3^ (large-volume group), respectively. The F98 glioma model is highly proliferative and has the characteristics of a high-grade glioma (Mathieu *et al.*, 2007 ▶).

For the small tumor groups (Table 1 ▶), 18 mice were injected with eGFP expressing F98 glioma cells. These cells were stably transfected with a plasmid expressing eGFP under the control of the CMV promoter (peGFP-N1 vector; Clontech Laboratories, Palo Alto, CA, USA).

### 2.3. Irradiation procedure   

#### 2.3.1. Animal preparation   

**Figure 1:**
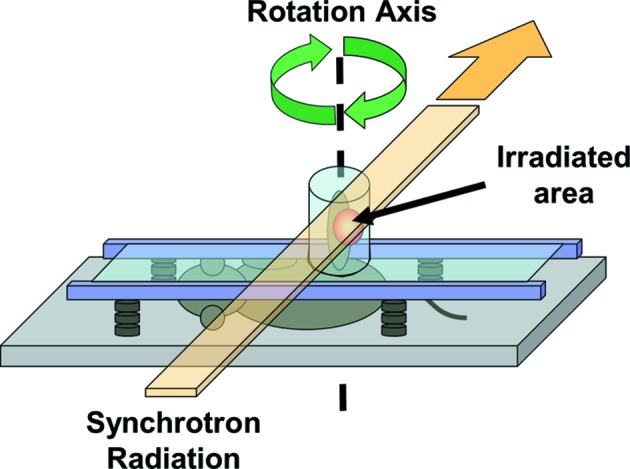
Irradiation set-up. Schematic representation of the positioning of the mouse in the horizontal frame with the left hindlimb maintained in a vertical plexiglas tube.

Animals were randomized into eight different groups (see Table 1 ▶). Twenty-four hours before the irradiation, 2.5 µg of cisplatin (Cisplatyl, Sanofi-Aventis, Paris, France) in 50 µl of a saline solution was injected manually into the center of the tumor with a 29 gauge needle for 30 s. In the study of Biston *et al.* (2004 ▶), the cisplatin distribution was homogeneous in the tumor 24 h after intra-tumoral injection as measured by X-ray fluorescence. The following day, animals were anaesthetized with an intraperitoneal injection of a mixture of 10 mg kg^−1^ xylazine and 100 mg kg^−1^ ketamine in a 0.9% NaCl saline solution. They were positioned on a home-made horizontal frame with the left hindlimb immobilized vertically in a plexiglass tube (Fig. 1 ▶). Their body temperature was maintained at physiological conditions during the whole experiment using heated gels.

#### 2.3.2. ESRF source   

A highly collimated and monochromatic synchrotron beam was used. The radiation source was a multi-magnet wiggler situated 150 m upstream of the animal. Divergence of the source was 0.1 mrad vertically and about 3 mrad horizontally. Collimation slits were set to define a fan beam of 1.5 mm × 40 mm. The beam was monochromated at 79 keV, just above the absorption *K*-edge of the platinum (78.39 keV). Monochromatization was achieved by means of a pair of bent silicon crystals in transmission (Laue) mode. Band-pass was approximately 80 eV.

The animals on the horizontal frame (see Fig. 1 ▶) were fixed on a rotation and translation motorized stage. The rotation axis was positioned in the middle of the tube so that with a 1.5 mm-high and 40 mm-wide beam the whole surface within the cylinder (diameter = 20 mm) was irradiated. Before each irradiation, radiography of the left hindlimb of the mouse was performed. The radiography helped to localize the tumor and to determine the area to be irradiated. Animals were irradiated in multiple slices 1.5 mm high while rotating in a tomographic mode in order to have a more homogeneous dose deposition in the tissues; the total height of the irradiated volume varied between 12 and 21 mm (eight to 14 joining slices of height 1.5 mm) depending on the size of the tumor. The dose rate was 9.896 × 10^−4^ Gy mA^−1^ s^−1^ as measured at the skin entrance inside the plexiglas tube (Fig. 1 ▶), normalized to the electron current of the synchrotron storage ring. The device rotation speed was adjusted to deliver 15 Gy to the center of rotation. This dose was chosen as it has proven to have the best effects on rats bearing F98 gliomas (Biston *et al.*, 2004 ▶; Rousseau *et al.*, 2007*a* ▶).

### 2.4. Tumor volume measurements   

The reference tumor volume of all animals in the different groups (*t* = 0) was measured 24 h before the irradiation whether the group was irradiated or not. After the irradiation, the tumor growth was regularly evaluated. Length *l* and width *w* of the tumor were measured using a caliper. The height of the tumor was not taken into account. This may overestimate tumor volume in groups 4 and 8, where in 15% of the cases a central collapse was observed. The volume *V* was calculated using the following equation: *V* = (4/3)π*lw* ^2^. The reference tumor volume was normalized to 100 in every graph of this paper. Statistical analyses were realised using *GraphPad Prism4* software (San Diego, USA). A Student’s unpaired two-tailed *t* test was used to compare the mean values. Data are presented as mean ± SEM (standard error of the mean) and differences between the means were considered to be statistically significant at a probability of *p* < 0.05.

### 2.5. Two-photon microscopy   

#### 2.5.1. Animal preparation   

Five days after the irradiation, mice were anesthetized with a continuous inhalation of isoflurane (2%) in a gas mixture of O_2_ and N_2_O (30% and 70%, respectively). The hindlimb of the mouse was blocked in a home-made restrain in order to reduce the breathing movements. Core temperature was maintained at approximately 310 K using warm water circulating through a pad. Skin (4–5 mm in diameter) was removed above the tumor. An image of the exposed area was recorded using a Pentax IstDL camera mounted on a macroscope (Leica MZ6).

**Figure 2:**
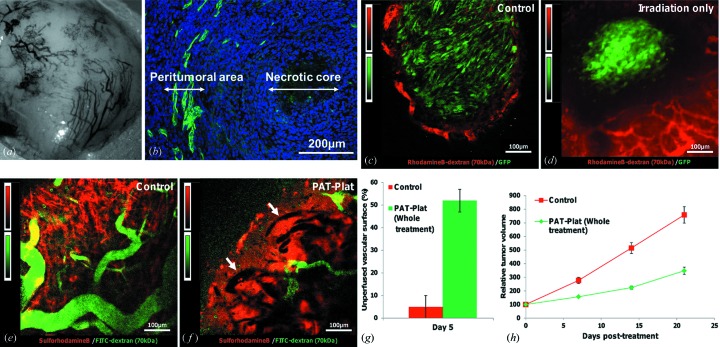
Effects of the treatment on tumor vasculature. (*a*) Macrophotography of the surface of the F98 glioma implanted in the hindlimb of a mouse after the removal of the skin. (*b*) Immunohistochemistry performed on a section in the middle of a F98 glioma. Green: immunolabelling of the collagen IV indicating the basal lamina of the vasculature in the peritumoral area; blue: cell nuclei stained with Hoechst 33342. Note the necrotic core at the center of the tumor. (*c*) Two-photon imaging of an untreated F98-GFP glioma at day 5 after the beginning of the study. Green: GFP-F98 glioma cells; red: rhodamineB-dextran 70 kDa dye staining of blood vessels. Only the tumor periphery is perfused until a maximum depth of approximately 100 µm. All two-photon images, (*c*)–(*f*), were taken at a depth of 50 µm beneath the surface of the tumor. (*d*) Two-photon imaging of a F98-GFP glioma at day 5 after receiving 15 Gy 79 keV tomographic synchrotron irradiation. Green: GFP-F98 glioma cells; red: rhodamineB-dextran 70 kDa dye staining the blood vessels. The tumor periphery is still perfused. The solid tumor mass decreased; see the empty space between functional red vessels and the green tumor cells. In comparison with the control tumors (*c*), cell morphology has changed from elongated shapes into round shapes. (*e*) Two-photon imaging of an untreated F98 glioma at day 5 after the beginning of the study. Green: vasculature labeled by FITC-dextran 70 kDa; red: sulforhodamineB dye extravascated in the extravascular extracellular tumor volume in order to obtain a better contrast. A rolling-ball background subtraction with a radius of ten pixels was applied (with this method, a local background value is determined for every pixel by averaging over a very large ball around the pixel; this value is then subtracted from the original image, removing variations of the background intensities). Note that for the staining of the functional vessels, FITC-dextran (green signal) instead of rhodaminB-dextran (red signal) is used. (*f*) Two-photon imaging of a F98 glioma at day 5 after the whole treatment. Note that the non-perfused vessels areas appear dark (see white arrows) and a few functional vessels show up green after perfusion with FITC-dextran surrounded by a red SRB staining of the whole extravascular volume. A rolling-ball background subtraction with a radius of ten pixels was applied. (*g*) Unperfused vascular surface (%) in control (group 1) and PAT-Plat (group 4) animals at five days post-treatment. (*h*) Tumor growth in control (group 1; red squares) and PAT-Plat (group 4; green diamonds) animals during the first three weeks post-treatment. The decrease of the perfused vascular surface observed in PAT-Plat animals (*g*) can be correlated with the reduction of the tumor growth.

Two different experiments were performed:

(i) *Estimation of the percentage of the non-perfused vascular surface area after PAT-Plat.* In F98 gliomas of the control group 1 and the whole treatment group 4, a cocktail of 100 µl of a 100 mg ml^−1^ FITC-dextran 70 kDa solution (Sigma-Aldrich) and 50 µl of a 10 mg ml^−1^ 0.56 kDa sulforhodamineB (SRB) solution (Lambda Physics) in 0.9% NaCl was injected into the tail vein. The freely diffusible SRB stained the extravascular volume and the perfused vessels of the tumor except the non-perfused vessels [see Figs. 2(*e*) and 2(*f*) ▶].

(ii) *Analysis of tumor cell morphology and tumor blood perfusion changes for all treatment modalities.* In F98-GFP gliomas (groups 5–8), the perfused vasculature was stained with 100 µl of a 100 mg ml^−1^ rhodamineB-dextran 70 kDa solution (Sigma-Aldrich). The numbers of GFP-F98 implanted animals are indicated in Table 1 ▶.

#### 2.5.2. Microscopy set-up   

Two-photon laser-scanning microscopy was performed with a Biorad MRC 1024 scanhead and an Olympus BX50WI microscope. Fluorescence signal was directly epicollected. An 800 nm excitation beam from a Tsunami femtosecond Ti:sapphire laser (5 W pump; Spectra-Physics, Millenia V) was focused onto the surface of the tumor using a 20× water-immersion objective (0.95 NA, Xlum Plan Fl Olympus). The beam was scanned in the *xy* plane to acquire 512 × 512 pixel images in 0.9 s between 0 to 200 µm below the surface. Variation of the observation depth (*z*-scan) was realised by moving the motorized objective vertically. The incident laser intensity was adjusted by using a half-wave plate and a polarizer placed before the microscope. Total average power delivered at the surface ranged from 1 to 150 mW. Two channels (red and green) could be simultaneously observed using two added external photomultiplier tubes (PMTs) equipped with an appropriate set of filters: HQ 620/60 and 710 ASP in front of PMT1 (red channel); HQ 535/30 and BG39 in front of PMT2 (green channel). Images were acquired using the Biorad exploitation system and displayed using *ImageJ* software (version 1.36, Public Domain Software, available from http://rsb.info.nih.gov/ij/).

#### 2.5.3. Measurement of the non-perfused vascular surface   

Quantification of the non-perfused vascular surface per surface of the whole tumor sections in the two-photon image was assessed on the merged image showing the perfused vessels (FITC-dextran, green) and the extravascular space after diffusion of SRB (red) (Fig. 2*f* ▶). Two-photon images were analyzed at a depth of 50 µm beneath the surface of the tumors. Note that only perfused tumor vessels were presented at the tumor periphery in the control group until a depth of maximum 100 µm (see Fig. 2*c* ▶). Note that in Figs. 2(*e*) and 2(*f*) ▶ we used FITC-dextran (green signal) instead of rhodamineB-dextran (red signal) to stain the functional vessels, because the freely diffusible red dye SRB was applied to stain the whole extravascular space.

After selection of the whole tumor surface area, in two-photon images like in Fig. 2(*f*) ▶ the merged color image was binarized and the percentage of the non-perfused black pixels was calculated as follows: [(all tumor surface area pixels − white pixels)/all tumor surface area pixels] × 100. The white pixels are the pixels that contained a fluorescence signal: whether FITC-dextran (green) in perfused vessels or SRB (red) in the extravascular space (see Fig. 2*f* ▶). After intra­venous injection of both dyes, the SRB diffused freely from functional tumor vessels inside the extravascular space of the tumor, but did not stain the lumen of non-perfused vessels that remained dark.

These calculations were performed for four mice at day 5 after the whole PAT treatment (group 4, Table 1 ▶) and for six control mice (group 1, Table 1 ▶). Data are presented as mean ± standard deviation.

### 2.6. Immunohistochemistry   

#### 2.6.1. Slice preparation   

Animals (control groups 1 and 5, *n* = 6) were sacrificed and their left hindlimbs were excised and frozen in isopentane at 193 K. Slices of thickness 10 µm were cut on a cryotome (Microm HM560) and conserved at 251 K until the histological and immunohistochemistry experiments.

#### 2.6.2. Immunohistochemistry   

For the staining of the basal lamina of all tumor vessels, sections were fixed in a solution of paraformaldehyde (4%) and then permeabilized with a lysis buffer: sucrose 300 m*M*, MgCl_2_ 3 m*M*, Tris 20 m*M*, NaCl 50 m*M* and Triton 0.5%. They were incubated overnight at 277 K with goat anti-collagen IV (1/100, Southern Biotech) in PBS-BSA 3%. Finally, sections were incubated during 90 min at room temperature with FITC conjugated donkey anti-goat (1/100, Jackson Immunoresearch) in PBS-BSA 3%. Slices were mounted with a mounting medium (Biomeda, Foster City) containing Hoechst 33342 (2 µg ml^−1^) for the staining of all nuclei and observed on a Nikon epifluorescence microscope (type Eclipse E600) connected to a digital color camera (Olympus, Color View II) and *analySIS* software (Version 5, Soft Imaging System, Olympus, Munster, Germany) for image acquisition and processing.

## 3. Results   

The effects of the different treatment modalities were analyzed, regarding tumor volume, tumor blood perfusion and glioma cell morphology on large and small tumor groups within 40 days after the start of a treatment. The rationale behind this choice was to study the consequences of the treatment at two different stages of glioma progression.

### 3.1. Treatment of large tumor volumes   

**Figure 3:**
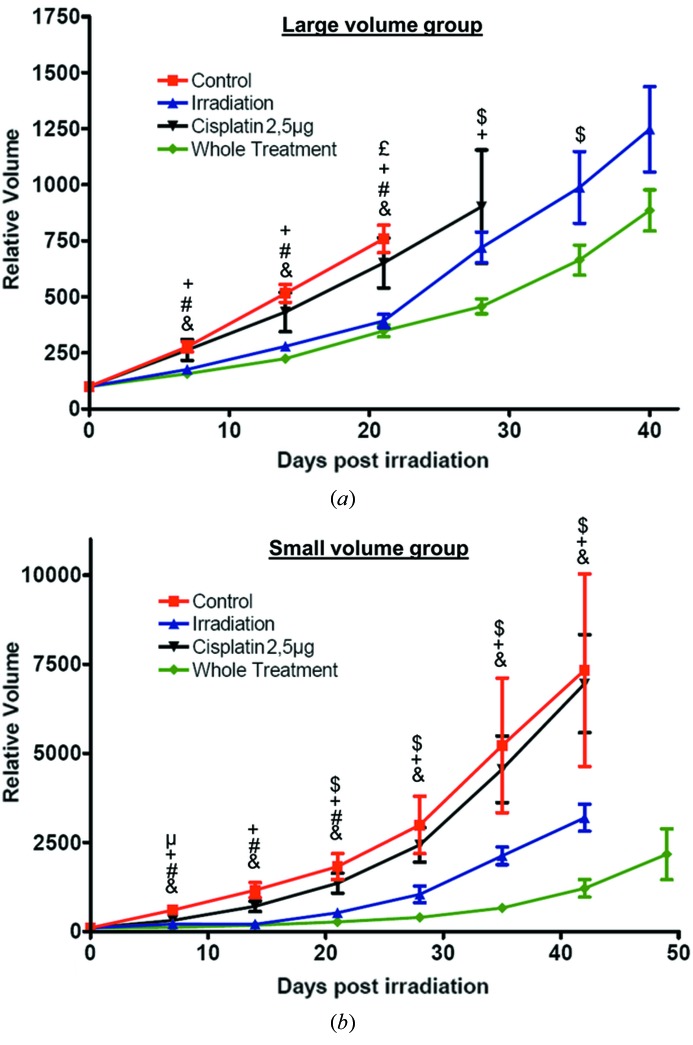
(*a*) Effects of the therapy on tumor growth for animals bearing a large tumor (mean volume 480 ± 33 mm^3^) at the moment of the treatment. Effects of the intratumoral injection of 2.5 µg of cisplatin alone (group 2; black down-triangles), the radiotherapy alone (group 3; blue up-triangles), the whole treatment with 2.5 µg of cisplatin (group 4; green diamonds) and no treatment (group 1; red squares) on the tumor growth. (*b*) Effects of the therapy on tumor growth for animals bearing a small tumor (mean volume 106 ± 11 mm^3^) at the moment of the treatment. Effects of the intratumoral injection of 2.5 µg of cisplatin alone (group 6; black down-triangles), the radiotherapy alone (group 7; blue up-triangles), the whole treatment with 2.5 µg of cisplatin (group 8; green diamonds) and no treatment (group 5; red squares) on the tumor growth. For each group the tumor volume was normalized to 100 at the moment of the treatment. &: *p* < 0.05 whole treatment *versus* control; #: *p* < 0.05 irradiation alone *versus* control; +: *p* < 0.05 whole treatment *versus* cisplatin alone; £: *p* < 0.05 irradiation alone *versus* cisplatin alone; $: *p* < 0.05 whole treatment *versus* irradiation alone; μ: *p* < 0.05 cisplatin alone *versus* control; unpaired two-tailed Student’s *t*-test.

Experiments were first conducted on tumors with a mean volume of 480 ± 33 mm^3^ at the start of the treatment. Intratumoral injection of cisplatin elicited no modifications of the tumor growth neither with 2.5 µg (Fig. 3*a* ▶) nor with 5 µg (results not shown). A synchrotron irradiation with a dose of 15 Gy and energy of 79 keV induced a decrease of the growth rate observable as early as one week after the irradiation (Fig. 3*a* ▶). More interestingly, the whole treatment (cisplatin + irradiation) led to a higher decrease of the growth rate in comparison with irradiation alone. The difference between irradiation alone and the whole treatment is significant starting at three weeks after the irradiation (Fig. 3*a* ▶). It must be noted that the volume decrease after the whole treatment is enhanced with an injection of 5 µg of cisplatin rather than with 2.5 µg (results not shown). However, systemic toxicity of the whole PAT-Plat protocol with a 5 µg injection of cisplatin was observed as all animals died approximately at 28 days post-irradiation. These systemic toxicity effects were not observed in the other groups of Table 1 ▶.

### 3.2. Treatment of small tumor volumes   

The same experiments were performed on tumors with a mean volume of 106 ± 11 mm^3^. A dose of 2.5 µg of cisplatin per tumor was used to avoid systemic toxicity effects as seen at a dose of 5 µg per tumor. The efficiency of the whole treatment (group 8) in comparison with irradiation only (group 7) was significant, starting from two weeks after irradiation (Fig. 3*b* ▶). This is one week earlier than observed for the large tumors. Moreover, the difference between these two groups remains significant at longer delays (42 days), which is not found in the large tumor group. Finally, cisplatin treatment alone still had no significant effects on the tumor volume (Fig. 3*b* ▶).

### 3.3. Effects on the tumor vasculature and cell morphology   

We have investigated the effects of the whole PAT-Plat treatment and its different modalities on tumoral vasculature to see whether the effects observed on tumor volume were possibly related to earlier modifications of the tumor blood perfusion.

Non-treated F98 glioma exhibit a necrotic core surrounded by a proliferative peritumoral area that is highly vascularized. Angiogenic vessels that were found in these areas are large and disorganized [Figs. 2(*a*) and 2(*b*)–2(*e*) ▶]. At five days after irradiation, in gliomas of group 7 (irradiation only, Fig. 2*d*), we observed a reduction of GFP-positive glioma cells at the periphery and a change in their cell shapes in comparison with the control group 5 (Fig. 2*c* ▶). They showed more round-shapes in comparison with elongated-shapes in the control group. Note that the vasculature at the tumor periphery [Fig. 2(*d*), red rhodamineB-dextran staining] was still perfused after irradiation, in comparison with gliomas of group 4 [Fig. 2(*f*) ▶, green FITC-dextran staining].

Five days after the whole treatment, many vessels were not perfused [see arrows in Fig. 2(*f*) ▶] in comparison with untreated controls (Fig. 2*e* ▶). The percentage of the unperfused vascular surface was 5 ± 5% in controls (group 1, Table 1 ▶) and 52 ± 5% in PAT-treated animals (group 4, Table 1 ▶) (Fig. 2*g* ▶). The percentage of the unperfused vascular surface could not be estimated for the small tumor groups 5 and 8, because red rhodamineB-dextran signals in the perfused vessels could not be separated from the intense red background in the two-photon images (see Fig. 2*d*). All these changes in tumor blood perfusion and cell shapes were observed before a decrease in tumor volume (Figs. 3 ▶ and 2*h* ▶). Tumor blood perfusion changes were only observed after the PAT protocol in group 4.

## 4. Discussion   

Up to now, effects of the PAT-Plat on the F98 glioma were essentially observed in terms of animal survival (Biston *et al.*, 2004 ▶; Rousseau *et al.*, 2007*a* ▶). In the present study, the effects of the PAT-Plat on the functional tumor vasculature followed by tumor volume changes were analyzed in time. For this purpose, a nude mouse model with F98 gliomas (Mathieu *et al.*, 2007 ▶) implanted in the hindlimb was used. This allowed longitudinal observations of the tumor volume without using imaging techniques such as magnetic resonance imaging. Moreover, it enables the observation of the vasculature in the peritumoral area (Bergers & Benjamin, 2003 ▶; Jain *et al.*, 2007 ▶) using intravital two-photon microscopy. Finally, in the glioma nude mouse model, the effect of T-cell mediated immune response can be avoided in the treatment response. We have chosen to test the chemo-radiotherapy treatment on two tumor groups with different volumes at the start of the treatment. The rationale behind this choice is to have an early stage of glioma development and a mature stage with a necrotic core.

Combination of chemotherapy using halogenated agents such as cisplatin and radiotherapy has shown to improve the prognosis of a large array of malignancies including head and neck, lung and cervical cancer (Bartelink *et al.*, 2002 ▶). Drug delivery was also considered as a major concern. Systemic i.v. infusion of the chemotherapeutic agent is often the source of important side effects (Muldoon *et al.*, 2007 ▶; Marshall *et al.*, 2006 ▶; Nakagawa *et al.*, 1993 ▶; Boulikas & Vougiouka, 2003 ▶) and poor drug accumulation in the tumor core. In our protocol, cisplatin was injected directly into the tumor 24 h before the irradiation in order to maximize the drug concentration in the tumor volume. In the study of Biston *et al.* (2004 ▶) the cisplatin distribution was homogeneous in the tumor 24 h after intra-tumoral injection as measured with X-ray fluorescence. We based our onset of irradiation on these observations. This time is long enough for diffusion inside the cells and cross-linking with DNA. At 24 h after cisplatin injection, we probably exclude free unbound cisplatin in the tumor.

Another method, convection enhanced delivery, may provide a better control of the injection and drug distribution in the tumor (Rousseau *et al.*, 2007*b* ▶).

In the following section, the effects of the therapy in terms of tumor volume and vascular modifications are discussed and a possible link between these two parameters is suggested.

### 4.1. Effects of the treatment on tumor growth   

#### 4.1.1. Effect of radiation only   

In the present paper, it was observed that a 15 Gy/79 keV irradiation decreased the tumor growth rate. Biston *et al.* (2004 ▶) and Adam *et al.* (2006 ▶) have both reported a median survival time of 26 days for rats implanted with 1000 F98 cells in the striatum. The median survival time was extended to 48 days and 46 days, respectively, after a 15 Gy irradiation. In our study, the tumor growth decrease was observed for three weeks following the irradiation, followed by an exponential increase. This decrease in tumor growth observed for three weeks (21 days) following the treatment is close to the increase survival observed by Biston *et al.* (2004 ▶) and Adam *et al.* (2003 ▶): 22 and 20 days, respectively.

Bencokova *et al.* (2007 ▶) classified F98 glioma cells as a moderate radiosensitivity cell line. They quantified the cell surviving fraction *in vitro* at 2 Gy to be 41.4 ± 2.5%. Despite this moderate radiosensitivity, the irradiation did not seem to sterilize the tumor cells (Fig. 2*d* ▶). In this figure we showed that a large amount of F98 glioma cells (GFP+) died but the whole tumoral volume was not sterilized. Recurrence occurred three weeks after the irradiation.

#### 4.1.2. Effects of the PAT-Plat   

After PAT-Plat, the tumor growth rate decrease was more important than after irradiation only. For the animals bearing a large-volume tumor at the moment of the treatment, regardless of the dose of cisplatin injected (2.5 or 5 µg), the difference between the irradiated group and the whole treatment group is significant starting from three weeks after the irradiation. For animals bearing a small-volume tumor at the moment of the treatment, this difference is significant starting from two weeks after the irradiation. However, animals were not cured.

These results differ from those of Biston *et al.* (2004 ▶), who have shown 33% cure of rats after the complete PAT-Plat therapy. The discrepancy can in part be explained by the role of the T-cell mediated immune response, which is present in the Fisher rat model but absent in the nude mouse model. The stimulation of the immune response after radiotherapy may partly account for tumor ablation. The immune system can have a central role in the response to tumor aggression. We hypothesize that the absence of animals showing a whole remission in this study can be accounted for by the absence of immune response.

We have observed that the reduction of the growth rate was more important for a 5 µg dose of cisplatin. However, the toxicity of the treatment under these conditions is high and, despite a stabilization of the growth curve, all animals died at day 28 post-treatment. Surprisingly, irradiation or 5 µg cisplatin alone did not induce an early death. We hypothesize that irradiation of cisplatin may produce radiolysis by-products of the latter, which may increase the systemic toxicity at higher concentrations of cisplatin and possibly participate in the synergetic effects of PAT (Gastaldo *et al.*, 2011 ▶).

In PAT-Plat studies on F98 glioma bearing rats (Biston *et al.*, 2004 ▶; Rousseau *et al.*, 2007*a* ▶) with a normal immune response, a distinction between additive or synergistic effects could not be made. In the present study, observations made on the various groups of animals have shown that cisplatin has no significant effects on the tumor growth; irradiation decelerates it and the whole treatment induces a decrease that is more important than the one observed with the irradiation alone. These findings suggest that the effect of cisplatin combined with irradiation is rather synergistic.

Biston *et al.* (2004 ▶) have hypothesized that the effects of the PAT-Plat could be explained by the emission of Auger electrons. When the platinum is irradiated above its *K*-edge energy, an electron of the *K*-shell is extracted creating a vacancy. This vacancy is filled by rearrangement of electrons from the outer shells leading to an Auger effect. Auger electrons interact at distances shorter than a few nanometers with target (macro) molecules. The targets can be the DNA as cisplatin binds to it. However, the most probable interaction is with the water molecules. Secondary electrons (photoelectrons, Auger electrons) can generate important quantities of reactive oxygen species (ROS) that can indirectly create DNA damage. These direct and indirect pathways create DNA single (SSB) and double (DSB) strand-breaks on tumor cells. Moreover, adducts of cisplatin on DNA can inhibit repair pathways after radiotherapy (Turchi *et al.*, 2000 ▶). In that case, the synergistic effect of the PAT-Plat is not only due to photoelectric effects but may be partly explained by the inhibition of DNA repair pathways.

### 4.2. Effects of the PAT-Plat on tumor vasculature   

Effects of the PAT-Plat on the tumor vasculature in the perfused rim of the gliomas were observed by means of intravital two-photon microscopy. At day 5 post-treatment (Fig. 2*f* ▶) a decrease of the perfused vascular surface at the periphery of the tumor was measured. These vessels are mostly located at the surface of the subcutaneous gliomas. These phenomena appeared largely before a regression of central tumor volume, which started three weeks after complete treatment.

We hypothesize that the whole treatment may induce endothelial cell death, collapse of the angiogenic vessels, thrombosis and consequently inhibition of tumor perfusion. Note that after irradiation only, the tumor vessels were still functional (Fig. 2*d* ▶).

**Figure 4:**
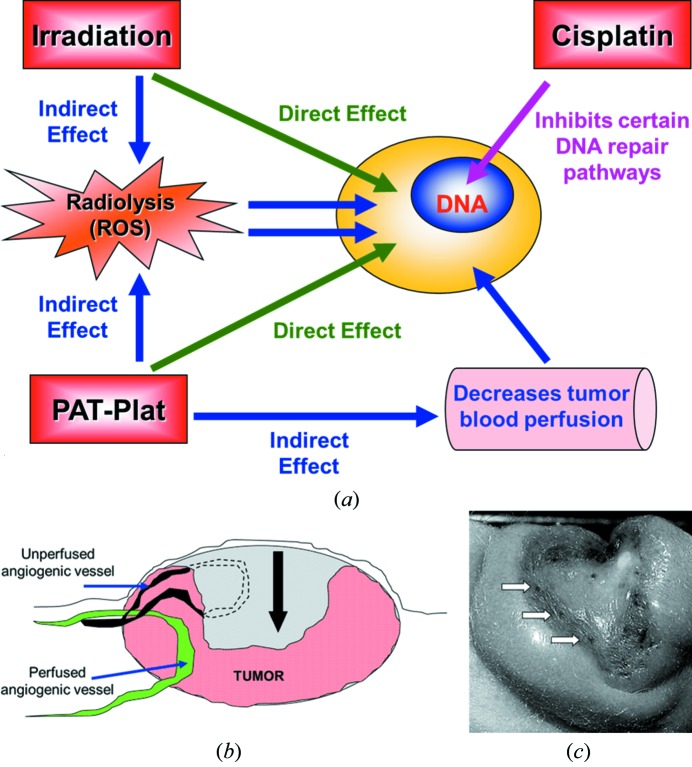
A hypothetical model explaining the effects of the PAT-Plat protocol. (*a*) General model for PAT-Plat effects. Irradiation leads to cellular damages such as DNA SSB and DSB directly or indirectly *via* the creation of reactive oxygen species. These damages are less likely repaired thanks to the inhibition of certain repair pathways by the cisplatin. Moreover, the PAT-Plat reduces the perfused vascular surface. This reduction of the perfusion can indirectly induce tumor cell death. (*b*) Schematic representation of the regression observed on a F98 glioma one month after the treatment. Grey area: part of the tumor that has disappeared after the whole treatment. The black arrow indicates the tumor volume reduction. (*c*) Macrophotograph of a F98 glioma one month after the whole treatment. Note that the dark spots show the blood vessel coagulations after the whole PAT-Plat treatment (white arrows).

In summary, we propose a model showing that photoactivation of cisplatin induces an effect on tumor cells mediated by DNA damage caused by photoelectrons and reactive oxygen species. More precisely, the irradiation and secondary electrons generated by the photoactivation of the cisplatin generate SSB and DSB on DNA that are less likely to be repaired as the cisplatin can inhibit some repair pathways (Turchi *et al.*, 2000 ▶). Moreover, a second effect is observed on proliferating vascular endothelial cells creating an inhibition of the tumor blood perfusion (Fig. 4*a* ▶). Comparable effects were observed on microvascular endothelium cells in the intestine after irradiation only (Paris *et al.*, 2001 ▶). The latter phenomenon had a larger impact on the hypoxic tumor regions [see Figs. 4(*b*)–4(*c*) ▶], which are less perfused than those at the periphery of the tumor. Here, glioma cells were close to the functional vasculature of the host muscle tissue, where tumor recurrence appeared first.

## 5. Conclusions   

The whole PAT-Plat protocol showed a synergistic effect between cisplatin and irradiation on the F98 glioma growth. In accordance with the present observations and the previous experiments using the PAT-Plat protocol (Biston *et al.*, 2004 ▶; Rousseau *et al.*, 2007*a* ▶), we hypothesize that the transient tumor growth reduction is synergistic interactions of tumor-cell-killing effects and reduction of the tumor blood perfusion. This preclinical study has shown that changes in tumor blood perfusion and consequently tumor volume are important parameters in the prediction of the tumor response after the PAT-Plat protocol.

## Figures and Tables

**Table 1:** Animals (*n* = 146) were randomized in eight groups depending on their tumor volume at the beginning of the therapy and the type of treatment

Experiment	Group	Type of treatment	Number of mice
Large volume (mean tumor volume: 480 mm^3^)	1	Control	14
	2	2.5 µg Cisplatin	7
	3	Irradiation	13
	4	Whole treatment	27

Small volume (mean tumor volume: 106 mm^3^)	5	Control	6 + 6 implanted with F98-GFP cells
	6	2.5 µg Cisplatin	7 + 3 implanted with F98-GFP cells
	7	Irradiation	5 + 3 implanted with F98-GFP cells
	8	Whole treatment	18 + 6 implanted with F98-GFP cells

5 µg Cisplatin		(Control, 5 µg cisplatin, irradiation, whole treatment)	31
